# Downregulation of SLC3A2 mediates immune evasion and accelerates metastasis in oral squamous cell carcinoma

**DOI:** 10.1111/jcmm.18010

**Published:** 2023-11-06

**Authors:** Jiang En Wu, You Li, Jun Hou

**Affiliations:** ^1^ Department of Oral and Maxillofacial Surgery The First Affiliated Hospital of Anhui Medical University Hefei China; ^2^ Pharmacology University College London London UK

**Keywords:** immune evasion, oral squamous cell carcinoma, SLC3A2

## Abstract

Oral squamous cell carcinoma (OSCC) is a common malignancy originating from oral mucosal tissue. OSCC cells employ immune evasion strategies to avoid immune attacks, but research on inhibiting immune evasion and delaying OSCC progression is limited. This study aimed to investigate how SLC3A2 downregulation mediates immune evasion and promotes metastasis in OSCC through bioinformatics analysis and cell experiments. Gene enrichment analysis was performed using human double sulphur death‐related genes from the GSEA database. Differentially expressed genes were selected from the GEO database. Diagnostic models were constructed and validated using gene expression datasets. Immune infiltration and function were analysed through Gene Ontology (GO) and Kyoto Encyclopedia of Genes and Genomes (KEGG) pathway enrichment analyses. Cell experiments were conducted to evaluate the impact of SLC3A2 on immune response in OSCC. Ten double sulphur death‐related genes were identified, with SLC3A2 and SLC7A11 being enriched in tongue squamous cell carcinoma‐related diseases. Differential expression analysis revealed five genes (SLC3A2, SLC7A11, RPN1, GYS1 and NDUFS1) of diagnostic significance. GO analysis showed enrichment in amino acid import and transmembrane transport, while KEGG pathway analysis highlighted enrichment in ferroptosis, diabetic cardiomyopathy, and Starch and sucrose metabolism. Experimental verification confirmed higher SLC3A2 expression in OSCC cells. Overexpression of SLC3A2 inhibited cell proliferation and reduced PD‐1 and CTLA‐4 expression. Reduced SLC3A2 expression in OSCC promotes immune evasion and tumour progression by impairing T lymphocyte function. This study provides insights into targeted regulation of SLC3A2 expression for immune response‐based therapies in OSCC.

## 1 INTRODUCTION

Oral squamous cell carcinoma (OSCC) is a common malignancy in the head and neck region, with over 300,000 new cases reported globally each year. It is more prevalent in males, with a male‐to‐female ratio of approximately 2:1. The incidence of OSCC is particularly high in certain regions, especially Asia and Eastern Europe.^1^, ^2^ The global incidence of oral cancer has been on the rise in recent decades, with OSCC accounting for the majority of cases.^3^, ^4^, ^5^ The incidence rates vary significantly between countries, with the highest rates concentrated in Asia, South America and the Western Pacific region. Unhealthy lifestyle habits, such as smoking, alcohol consumption and poor diet, are considered major risk factors for the development of OSCC. The occurrence and progression of OSCC are regulated by a series of genetic mutations and aberrant molecular pathways, involving dysregulation of multiple biological processes (BPs) such as cell proliferation, apoptosis, matrix adhesion, cell adhesion and inflammation.^6^, ^7^

Double sulphur death refers to a cell death mechanism triggered by various chemical substances. Double sulphur compounds, such as carbon disulphide and sulphur dichloride, can induce cell death by interfering with multiple BPs within the cell.^8^, ^9^ These processes include oxidative stress, mitochondrial damage, opening of mitochondrial channels and disruption of electron transport chain. Through these mechanisms, double sulphur compounds can lead to the release of cytochrome c, reduction of mitochondrial membrane potential, decrease in ATP levels and activation of apoptotic signalling cascade.^10^, ^11^ However, there is currently limited research exploring the relationship between double sulphur death and immune evasion in OSCC. Thus, further studies are needed to investigate the link between double sulphur death and immune evasion in OSCC.

SLC3A2 is a gene encoding the large neutral amino acid transporter 2 (LAT1), which plays a crucial role on the cell membrane. It is involved in the regulation of disulphide generation and metabolism. SLC3A2 modulates the levels of peroxides in the tumour microenvironment, thereby influencing the formation and dissociation of intracellular disulphide bonds. It has been found that the expression of SLC3A2 is positively associated with increased disulphide bond formation between tumour cells, promoting their invasiveness and metastatic ability.^12^ However, the regulation of OSCC by the mechanism of double sulphur death is not well understood, and further research is needed to explore the reciprocal regulation between the two.

It has been demonstrated that SLC3A2 expression levels are associated with remodelling of the cancer immune microenvironment and a number of immune checkpoints.^13^ Moreover, the regulatory role of SLC3A2 expression in double sulphur death and its potential to promote immune evasion in OSCC cells are currently not fully investigated. Therefore, this study aims to explore the molecular mechanisms by which the downregulation of SLC3A2 expression inhibits immune evasion and accelerates the progression of OSCC through the induction of double sulphur death.

## 2 MATERIALS AND METHODS

### 2.1 Enrichment analysis of genes associated with double sulphur death

A list of human genes related to double sulphur death was collected and downloaded from the GSEA public database (https://www.gsea‐msigdb.org/gsea/index.jsp, 28 July 2023). The DOSE R package was used to map these target genes to disease databases. The Kolmogorov–Smirnov test and hypergeometric test were employed to calculate the enrichment of target genes in OSCC. Functional enrichment analysis was performed using annotation tools such as DAVID (https://david.ncifcrf.gov/, 12 July 2023), with a focus on Gene Ontology (GO) terms related to double sulphur death in terms of BPs, molecular functions (MF), and cellular components (CC). Statistical analysis using Fisher's Exact test was conducted. In addition, enriched pathways were identified and validated using the KEGG PATHWAY database (https://www.kegg.jp/kegg/, 28 July 2023), and the association between target genes and these pathways was evaluated using the hypergeometric test.

### 2.2 Identification of double sulphur death‐related targets in oral squamous cell carcinoma

Gene expression microarray data related to ‘Oral squamous cell carcinoma’ was downloaded from the Gene Expression Omnibus (GEO) database (https://www.ncbi.nlm.nih.gov/geo/, 11 July 2023). The search criteria included keywords ‘Oral squamous cell carcinoma’ and the requirement for human samples. The GSE37991 dataset was selected, which consisted of two sample groups: 40 OSCC patients and 40 normal tissue samples. The bioconductor R package in R software was used to perform background correction, normalization, and calculation of expression values for the microarray data. The curated OSCC mRNA expression matrix was merged with the double sulphur death‐related gene expression matrix. The limma R package was then used to identify differentially expressed genes (DEGs) associated with double sulphur death between the OSCC group and the normal tissue group. The criteria for DEG selection were: adjusted *p* < 0.05 and |log2FC (fold change)| > 0.58. Heatmaps and clustering analysis were generated using the heatmap package. Furthermore, enrichment analysis of the DEG set related to double sulphur death was performed using ssGSEA and GSEA methods to identify enriched biological pathways and gene sets. The proportion of immune cells and the expression levels of immune‐related genes in OSCC samples were evaluated using common methods such as CIBERSORT, TIMER and ESTIMATE, and correlated with the enrichment results of the DEG set associated with double sulphur death.

### 2.3 Development of a lasso diagnostic model for core genes in double sulphur death and external independent validation

The GSE37991 dataset was used as the internal dataset for developing the diagnostic model of OSCC. Another gene expression matrix dataset related to OSCC was downloaded from GEO as the external validation dataset, which included transcriptome expression matrix files. Based on the previously selected core double sulphur death targets, a lasso diagnostic model was constructed using the aforementioned expression matrix data. The model was trained to obtain the best diagnostic model. Cross‐validation and internal validation were performed to select the lasso model with the best predictive ability. The core genes associated with double sulphur death in OSCC were used to generate ROC curves using R packages such as survival, caret, glmnet, survminer and survival ROC. Further analysis on the correlation and interactions of the core genes was conducted using R packages corrplot, circlize, limma and Performance Analytics.

### 2.4 Immune infiltration and immune function analysis of core genes in oral squamous cell carcinoma

The expression matrix data of OSCC obtained previously was subjected to deconvolution analysis using the CIBERSORT package. This algorithm allows estimation of cell composition in complex tissues based on standardized gene expression data and quantifies the abundance of specific cell types. The CIBERSORT package was used to generate the expression matrix of immune infiltrating cells related to OSCC. The immune cell sorting was performed using the immune selection Perl script. Background correction, normalization and expression value calculation for the microarray data were conducted using the limma R package. The CIBERSORT package was then utilized to estimate the cellular composition of OSCC and normal tissues. Bar charts were generated to analyse the composition of immune cells in each sample, and heatmap plots were created using the pheatmap package to visualize the distribution of immune cells. The corrplot package was employed to analyse the interactions between immune cell clusters in OSCC and to generate co‐expression plots of immune cell infiltration. The expression levels of immune cells in OSCC tissues and normal tissues were analysed using the vioplot package, and violin plots were generated. The immune‐related functions of DEGs associated with double sulphur death in OSCC were analysed using packages such as limma, GSVA, GSEABase, pheatmap and reshape2, aiming to achieve precision treatment.

### 2.5 Gene Ontology (GO) analysis of biological functions and KEGG pathway enrichment

The gene targets associated with double sulphur death in ‘Oral squamous cell carcinoma’ that are clinically significant were subjected to GO analysis using the clusterProfiler GO.R package in R software. GO analysis aims to describe the functions of gene products, including CC, MF and BP. The clusterProfilerKEGG.R package was used for KEGG pathway enrichment analysis, and the corresponding signalling pathway diagrams were generated using the pathview package. Enrichment factor values were analysed to assess the enrichment level of core pathways and explore the potential biological functions and signalling pathway mechanisms related to double sulphur death in OSCC.

### 2.6 Materials

Normal human oral epithelial cells (HOECs) and human OSCC SCC‐9 cells were purchased from Company X. DMEM high‐glucose culture medium, RPMI 1640 culture medium, and fetal bovine serum were purchased from Company Y. 0.25% trypsin–EDTA, SDS‐PAGE gel preparation kit, Western and IP cell lysis buffer were purchased from Company Z. CCK‐8 cell proliferation assay reagent was purchased from Company A. GAPDH antibody, SLC3A2, PD‐L1 and CTLA‐4 antibodies were purchased from Company B. Annexin V‐FITC cell apoptosis assay kit was purchased from Company Z. SLC3A2 overexpression plasmid was purchased from Company C.

### 2.7 Cell culture and transfection

Normal HOECs and human OSCC SCC‐9 cells were cultured in RPMI 1640 medium containing 10% FBS and 1% penicillin–streptomycin solution. The cells were grown in a cell incubator at 37°C with 5% CO2. When the cell density reached around 90%, the cells were passaged and the passaged cells were used for subsequent experimental validation. Recombinant lentiviral vectors overexpressing SLC3A2 and empty vectors were constructed. SCC‐9 cells were incubated in lentiviral medium containing polybrene (10 μg/mL) for 48 h, and then the viral medium was replaced with fresh RPMI 1640 medium containing 10% fetal bovine serum. After transfection for 72 h, cells were selected using 2 μg/mL puromycin to establish cell lines with stable overexpression of SLC3A2. The cell groups were as follows: HOEC cell group, SCC‐9 cell group, ove‐SLC3A2 SCC‐9 cell group and ove‐NC SCC‐9 cell group.

### 2.8 Cell proliferation assay

The cell viability of each group was measured using the CCK‐8 cell proliferation assay. Cells were seeded in a 96‐well plate at a density of 2 × 104 cells per well with 100 μL complete RPMI 1640 medium. After 24 h, the supernatant was removed and the cells were incubated in RPMI 1640 medium containing 10 μL CCK‐8 solution at 37°C with 5% CO2 for 1–4 h. The optical density was measured at a wavelength of 450 nm using a microplate reader.

### 2.9 Flow cytometry detection of PD‐1 and CTLA‐4

PD‐1 (Programmed cell death protein 1) and CTLA‐4 (Cytotoxic T‐lymphocyte‐associated protein 4) are two crucial immune checkpoint molecules that play a significant role in tumour immune evasion. PD‐1 is an immune checkpoint molecule expressed on activated T cells. When PD‐1 binds to its ligands, PD‐L1 or PD‐L2, it inhibits signal transduction in T cells, suppressing their activation and proliferation, thereby attenuating the immune response. On the other hand, CTLA‐4 is a cell surface protein expressed on immune cells. By competitively binding to its common ligands, CD80/CD86, CTLA‐4 inhibits signal transduction in activated T cells, suppressing the activity of cytotoxic T lymphocytes and dampening the immune response. Thus, detecting the expression levels of PD‐1 and CTLA‐4 is valuable for evaluating the sensitivity of patients to immune therapy, assessing the tumour's immune evasion mechanism and monitoring treatment efficacy. Cells from each group were collected by centrifugation at 1000 rpm for 5 min. After cell counting, 1 × 10^5^ cells were taken and washed twice with PBS. The cells were resuspended in 100 μL PBS and incubated with 1 μL PD‐1 and CTLA‐4 flow cytometry antibodies. After gentle mixing, the cells were incubated at room temperature in the dark for 15 min. The cells were washed once with PBS to remove nonspecifically bound antibodies. Then, the cells were resuspended in 200 μL PBS and transferred to flow cytometry tubes for fluorescence intensity measurement using a flow cytometer. Data analysis was performed using CytExpert software.

### 2.10 qRT‐PCR

Total RNA was extracted from cells using Trizol reagent (1 mL per well), followed by incubation in a 1.5 mL EP tube for 10 min. Then, 200 μL chloroform was added to each tube, followed by centrifugation at 4°C, 12,000 rpm for 15 min. The upper aqueous phase was transferred and mixed with 400 μL isopropanol. After multiple centrifugation steps, the supernatant was discarded and the precipitate was dissolved in 20 μL DEPC water. The cDNA was synthesized by reverse transcription under the following conditions: 25°C for 5 min, 50°C for 15 min, 85°C for 5 min and 4°C for 10 min. After dilution of the cDNA by 10 times, real‐time fluorescence quantitative PCR amplification was performed according to the reaction system. The primer sequences for qPCR synthesis are listed in Table 1.

**TABLE 1:** Primer sequences.

Gene	Forward primer sequence	Reverse primer sequence
GAPDH	5′‐TGAAGGTCGGAGTCAACGGATTTGG‐3′	5′‐TGATGGCATGGACTGTGGTCATGAG‐3′
PD‐1	5′‐CAAAAGGTGGAGTTTGCTGGA‐3′	5′‐ACTCCAGGCTGACATCTCCAC‐3′
CTLA‐4	5′‐AGTTTCATGCCCAGGTTTCCAGCC‐3′	5′‐CTGCTCCAGCACATCCAGCTTCAC‐3′
SLC3A2	5′‐GCACTCCATGGGAGACTTTGTC‐3′	5′‐AGAAAGCTGTCAGTGGCATCTC‐3′

### 2.11 Western blotting

Cells from each group were lysed with RIPA buffer, and the protein concentration was determined using the BCA method. Each well was loaded with 25 μg of total protein for protein SDS‐PAGE electrophoresis and then transferred to a PVDF membrane. The membrane was blocked with 50 g/L skim milk for 1 h, followed by incubation with primary antibodies against SLC3A2 (1:500), PD‐L1 (1:100), and CTLA‐4 (1:500) overnight at 4°C. After washing the membrane with TPBS, secondary antibodies were added and incubated on a shaker at 37°C for 2 h. The protein expression was detected using the DNR Bio‐Imaging System, and the relative expression levels of proteins in each group were calculated using Image J software.

### 2.12 Statistical analysis

Each experiment was repeated three times. Data analysis was performed using GraphPad Prism 8.0.1 and SPSS 25.0 software. The results were presented as the mean ± standard deviation. Differences among multiple groups were compared using anova, and differences between two groups were compared using *t*‐tests. A *p* < 0.05 was considered statistically significant.

## 3 RESULTS

### 3.1 Enrichment of disulphide death‐associated genes in OSCC

**FIGURE 1:**
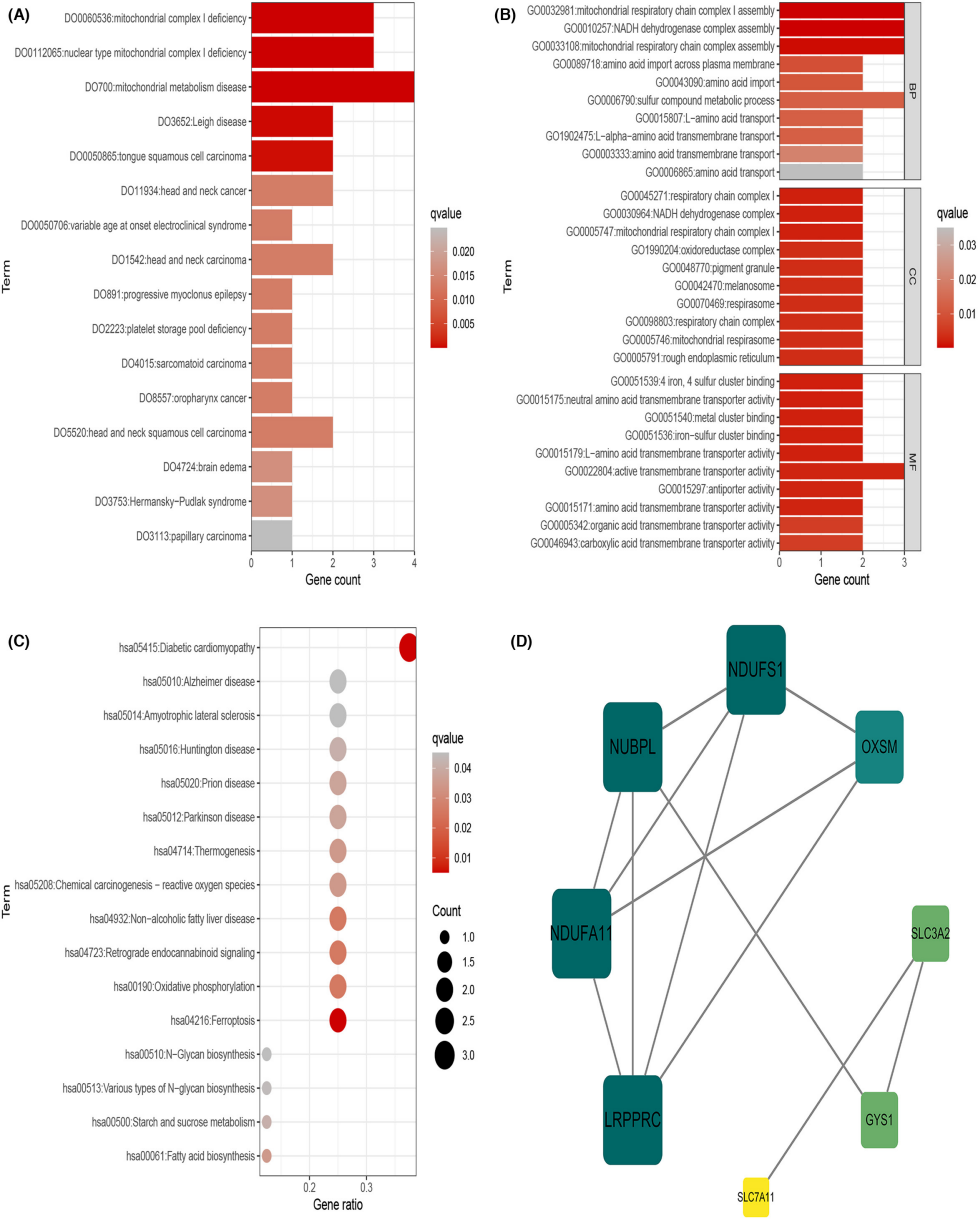
Enrichment analysis of genes associated with double sulphur‐induced cell death. (A) Enrichment analysis of differentially expressed genes (DEGs) using Gene Ontology (GO) terms. (B) Enrichment analysis of DEGs using Kyoto Encyclopedia of Genes and Genomes (KEGG) pathways. (C) Enrichment analysis of DEGs using protein–protein interaction (PPI) network analysis. (D) Core PPI network analysis of DEGs.

Ten disulphide death‐associated genes were obtained from GSEA analysis. The disease ontology (DO) analysis revealed that two genes, including SLC3A2 and SLC7A11, were enriched in OSCC‐related diseases. GO analysis showed enrichment in NADH dehydrogenase complex assembly, mitochondrial respiratory chain complex I assembly, and mitochondrial respiratory chain complex assembly. Kyoto Encyclopedia of Genes and Genomes (KEGG) analysis showed enrichment in ferroptosis, diabetic cardiomyopathy and oxidative phosphorylation (Figure 1A–C). A protein–protein interaction (PPI) network was constructed using these ten genes in the STRING database and further analysed using Cytoscape 3.7.2 software to identify the core PPI network (Figure 1D).

### 3.2 Differential expression analysis of disulphide death‐associated genes in OSCC

**FIGURE 2:**
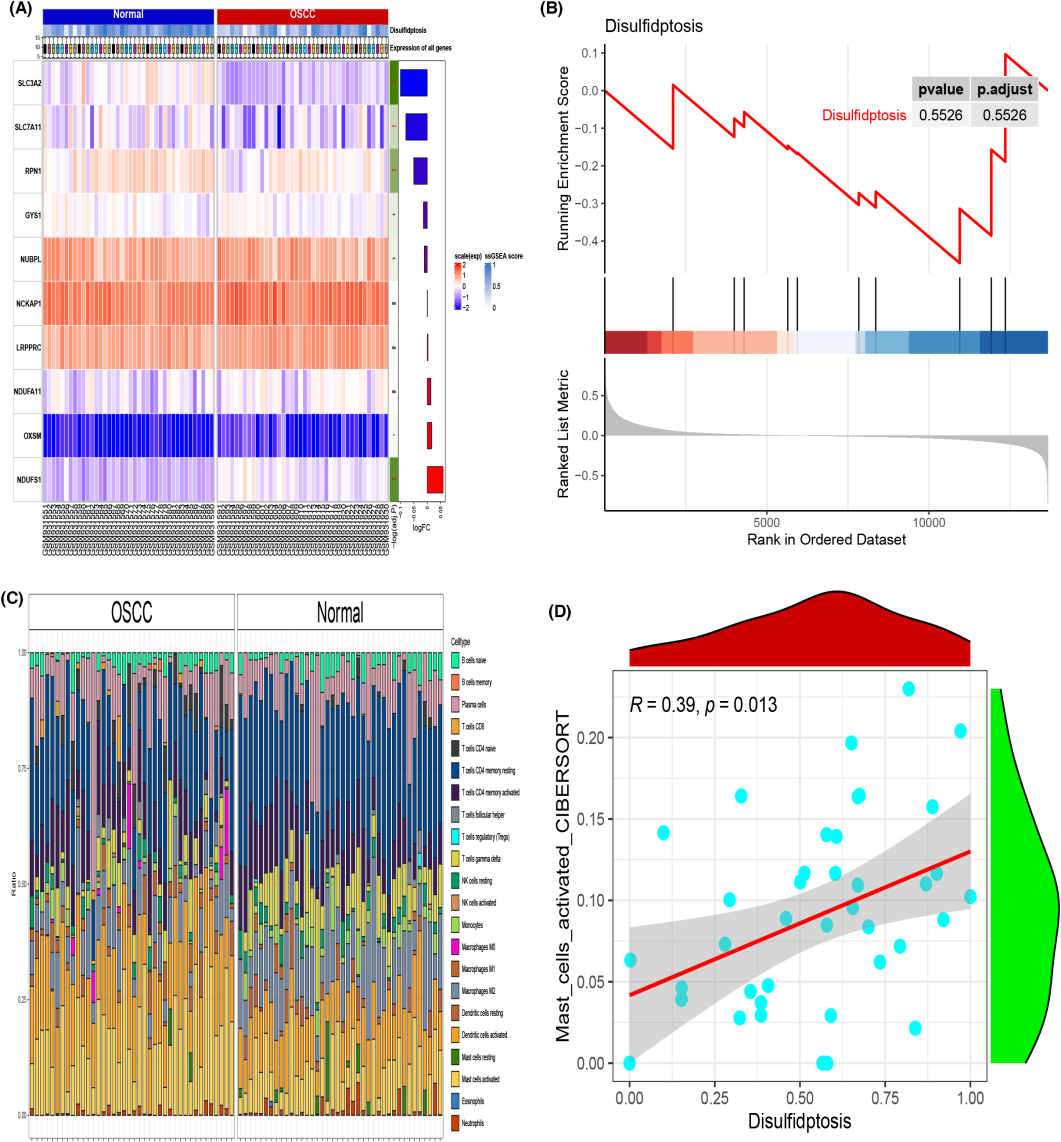
Differential analysis of genes associated with double sulphur‐induced cell death in oral squamous cell carcinoma. (A) Heatmap representing the expression patterns of differentially expressed genes (DEGs). (B) Gene set enrichment analysis (GSEA) revealing enriched pathways and gene sets in DEGs. (C) Distribution map of immune cells in the tumour microenvironment. (D) Correlation analysis between genes associated with double sulphur‐induced cell death and immune cell infiltration.

Using a criteria of *p* < 0.05 and fold change ≥1.50 (|log2FC| ≥ 0.58), five DEGs, namely SLC3A2, SLC7A11, RPN1, GYS1 and NDUFS1, were identified in the GSE37991 dataset. Heatmaps were generated to visualize the differential expression of these DEGs (Figure 2A). GSEA analysis revealed significant enrichment of the GSE37991 OSCC gene set in disulfidptosis compared to normal tissues (Figure 2B). The expression matrix data was background‐corrected, normalized and calculated using the limma R package in R. The CIBERSORT package was used to estimate the composition of immune cells in different groups. A bar graph was then generated to analyse the immune cell composition in each sample (Figure 2C). Additionally, significant correlations were observed between disulphide death‐associated genes and Mast cells activated and Eosinophils (Figure 2D).

### 3.3 Construction of diagnostic model

**FIGURE 3:**
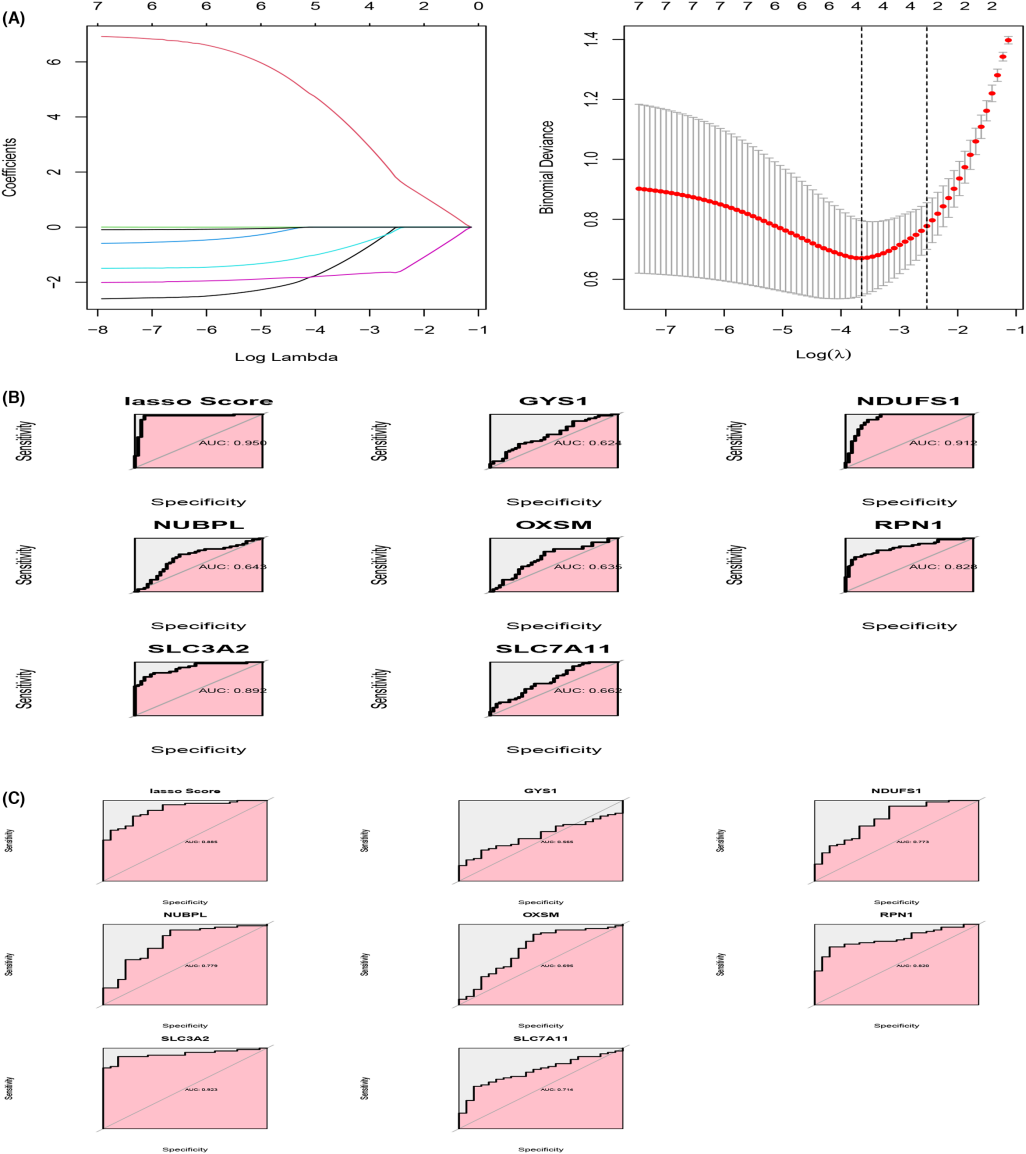
Construction of diagnostic model. (A) Lasso diagnostic model. (B) ROC curve for double sulphur‐induced cell death in the training set. (C) ROC curve for double sulphur‐induced cell death in the testing set.

The GSE37991 dataset was used as the internal dataset, and the GSE25099 dataset was used as the testing set to validate the reliable diagnostic model of disulphide death genes for OSCC. The lasso diagnostic model is shown in Figure 3A. Furthermore, ROC curve analysis was performed to evaluate the sensitivity and specificity of the expression changes of the aforementioned disulphide death‐associated DEGs in diagnosing OSCC. Figure 3B,C show the ROC curves. From Figure 3B, it can be observed that the AUC of NDUFS1, RPN1 and SLC3A2 in the GSE37991 dataset is greater than 0.70, indicating good diagnostic significance. In addition, Figure 3C shows that NDUFS1, RPN1, SLC3A2, NUBPL and SLC7A11 also have good diagnostic significance in the GSE25099 dataset, with AUC values all greater than 0.70.

### 3.4 Disulphide death‐associated gene network and immune function analysis in OSCC

**FIGURE 4:**
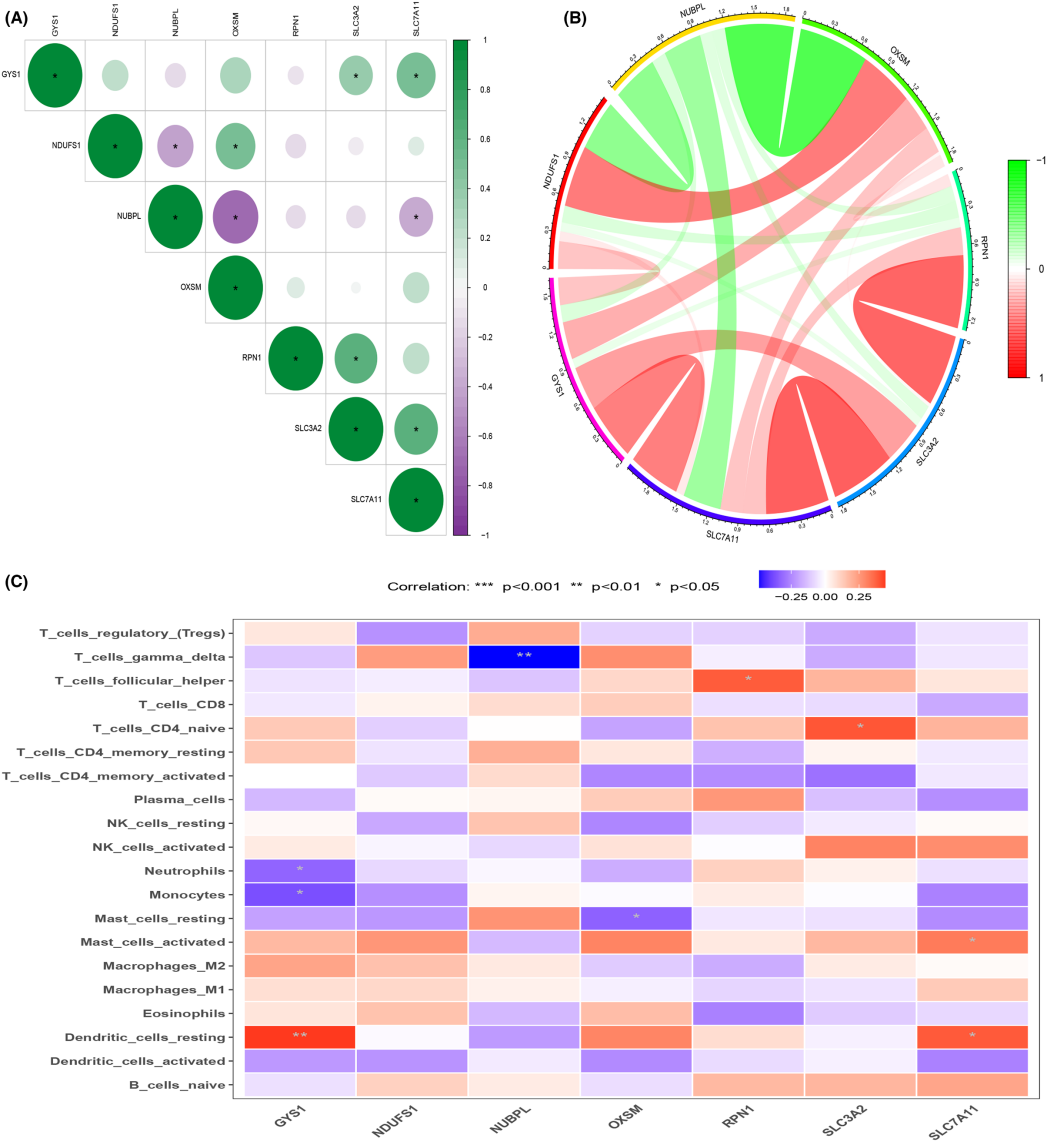
Gene interaction network and immune function analysis related to double sulphur‐induced cell death. (A) Interactions among genes associated with double sulphur‐induced cell death. (B) Gene interaction network associated with double sulphur‐induced cell death. (C) Correlation analysis between genes associated with double sulphur‐induced cell death and immune cell function.

Gene interaction networks were constructed based on the expression of disulphide death‐associated genes in the GSE37991 dataset. Positive correlations were observed between SLC3A2 and SLC7A11, RPN1, GYS1 (Figure 4A,B). Additionally, significant differences were found in the correlation between disulphide death‐associated genes and different immune cells (Figure 4C).

### 3.5 GO and KEGG enrichment analysis results

**FIGURE 5:**
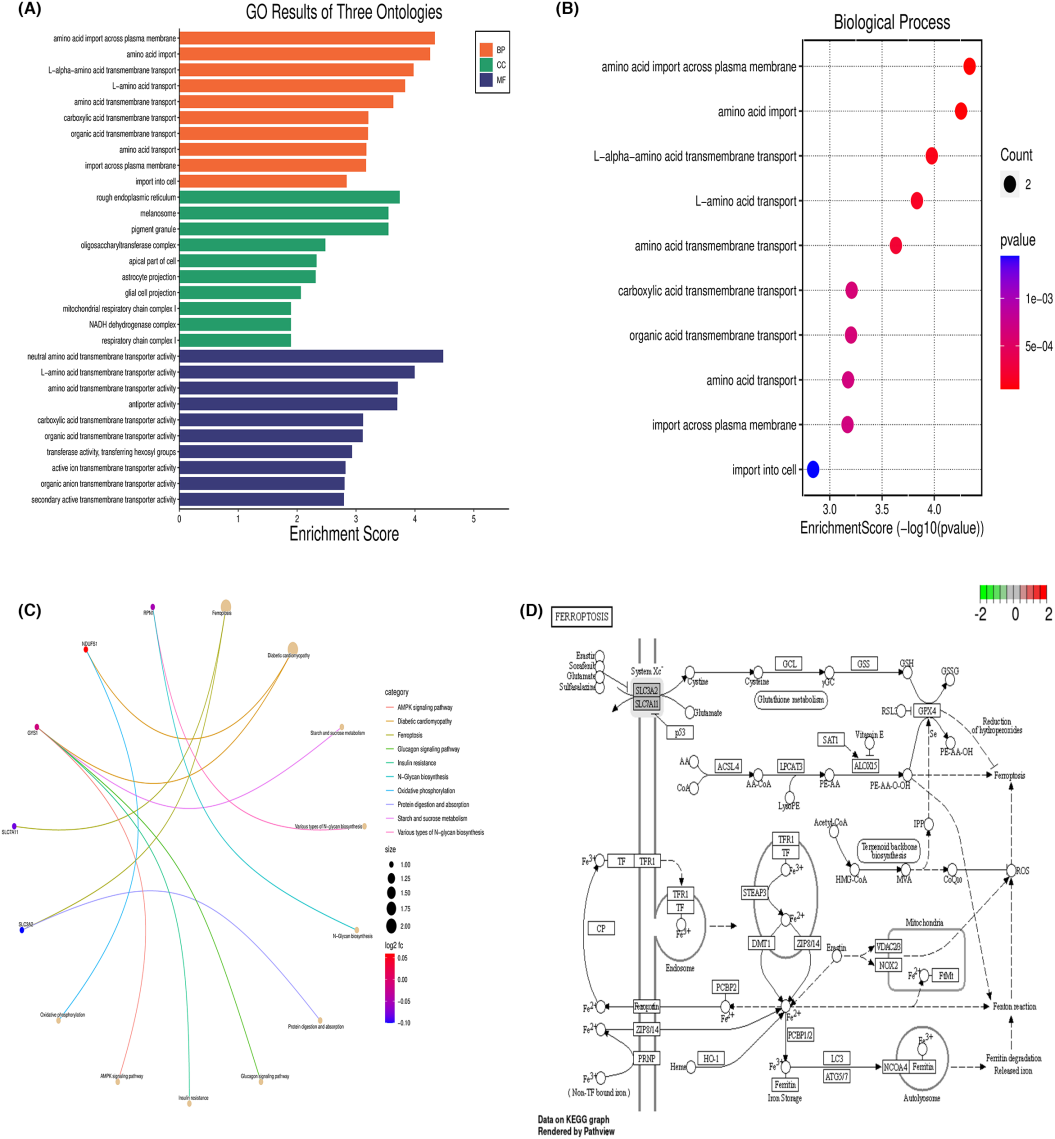
Enrichment analysis. (A) Bar graph depicting Gene Ontology (GO) functional analysis. (B) Chord diagram illustrating GO enrichment analysis. (C) Chord diagram showcasing Kyoto Encyclopedia of Genes and Genomes (KEGG) enrichment analysis. (D) Ferroptosis signalling pathway.

Using the Bioconductor and clusterProfiler packages in R, GO and KEGG pathway enrichment analysis were conducted for the five potential target genes associated with disulphide death in OSCC. The results of the potential target gene GO analysis showed enrichment in BPs such as amino acid import across the plasma membrane, amino acid import and L‐alpha‐amino acid transmembrane transport. For CC, enrichment was observed in the rough endoplasmic reticulum, melanosome and pigment granule. In terms of MF, enrichment was found in neutral amino acid transmembrane transporter activity, L‐amino acid transmembrane transporter activity and amino acid transmembrane transporter activity (Figure 5A,B). KEGG pathway enrichment analysis revealed significant enrichment in ferroptosis, diabetic cardiomyopathy, and Starch and sucrose metabolism (Figure 5C,D).

### 3.6 Decreased expression of SLC3A2 in OSCC cell lines

**FIGURE 6:**
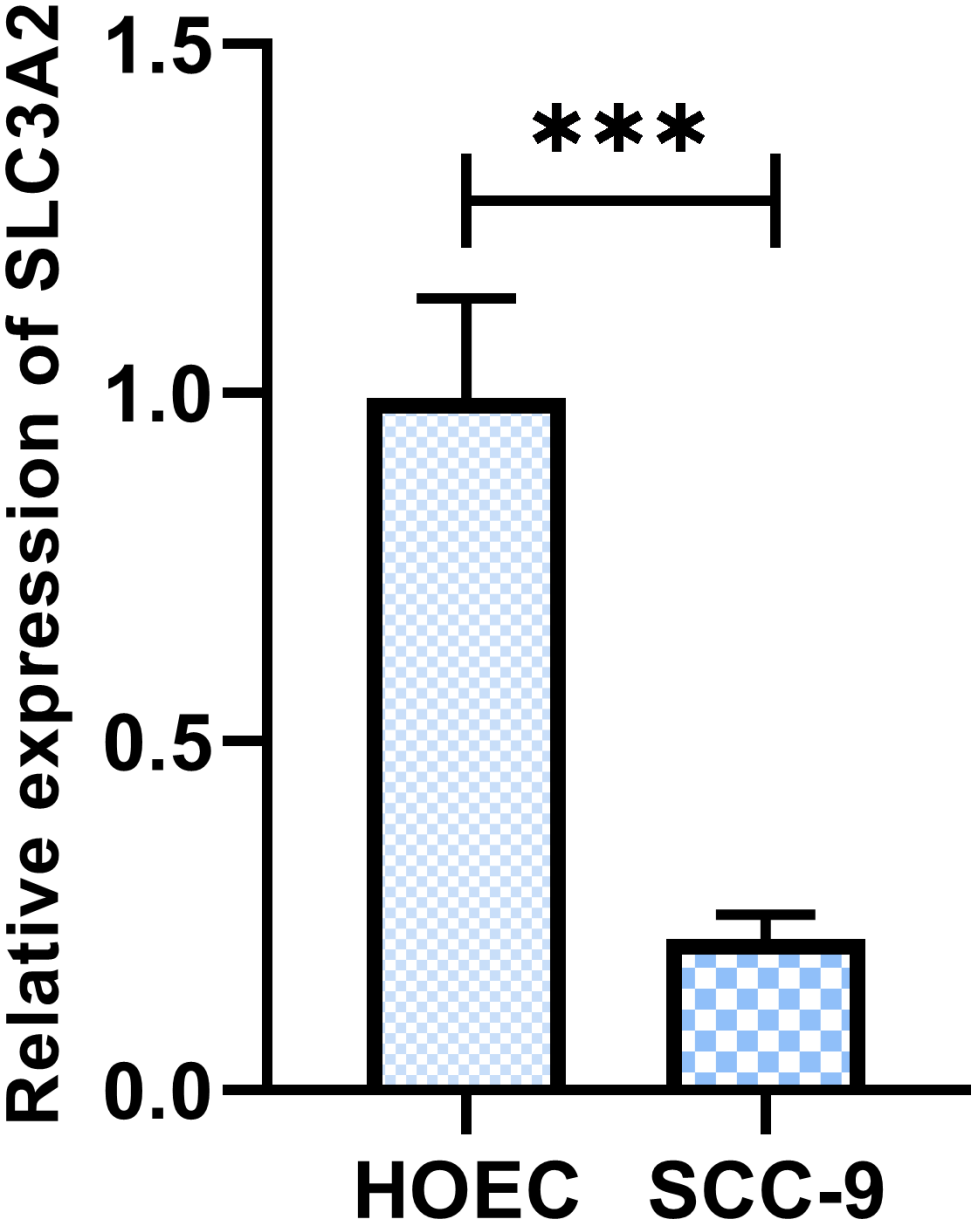
Demonstrates the downregulation of SLC3A2 expression in OSCC cell lines. ***p < 0.001.

RT‐qPCR was used to analyse the expression of SLC3A2 mRNA in SCC‐9 OSCC cell line and normal HOECs. The results showed that the expression level of SLC3A2 mRNA was higher in the SCC‐9 OSCC cell line compared to HOEC (*p* < 0.05, Figure 6). Lentivirus‐mediated overexpression of SLC3A2 was performed to create stable over‐SLC3A2 cell lines in SCC‐9 cells. RT‐qPCR was used to detect the transfection efficiency of lentivirus, and the results showed that the expression level of SLC3A2 was significantly increased in the over‐SLC3A2 group compared to the over‐NC group (*p* < 0.01). These results indicate the successful construction of stable over‐SLC3A2 cell lines for subsequent experimental studies.

### 3.7 Inhibition of cell proliferation by over‐SLC3A2 in OSCC cells

**FIGURE 7:**
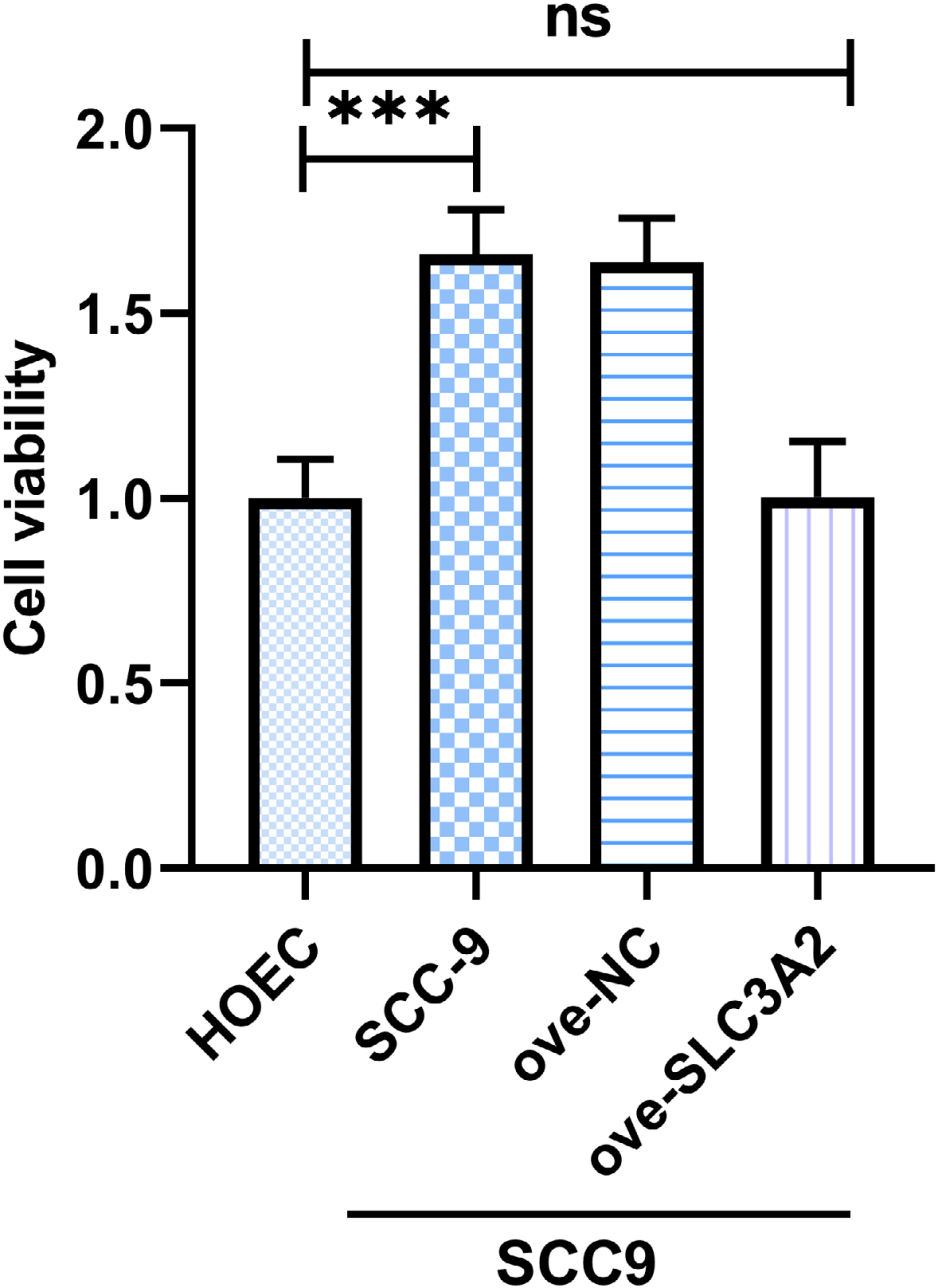
Inhibition of OSCC cell proliferation activity by overexpressed SLC3A2. ***p < 0.001.

To investigate the effect of over‐SLC3A2 on cell proliferation in SCC‐9 OSCC cells, CCK‐8 assays were performed. The results showed that overexpression of SLC3A2 significantly inhibited cell proliferation compared to the over‐NC group (*p* < 0.01, Figure 7). These results indicate that overexpression of SLC3A2 effectively inhibits cell proliferation in SCC‐9 OSCC cells.

### 3.8 Suppression of PD‐1 and CTLA‐4 expression by over‐SLC3A2

**FIGURE 8:**
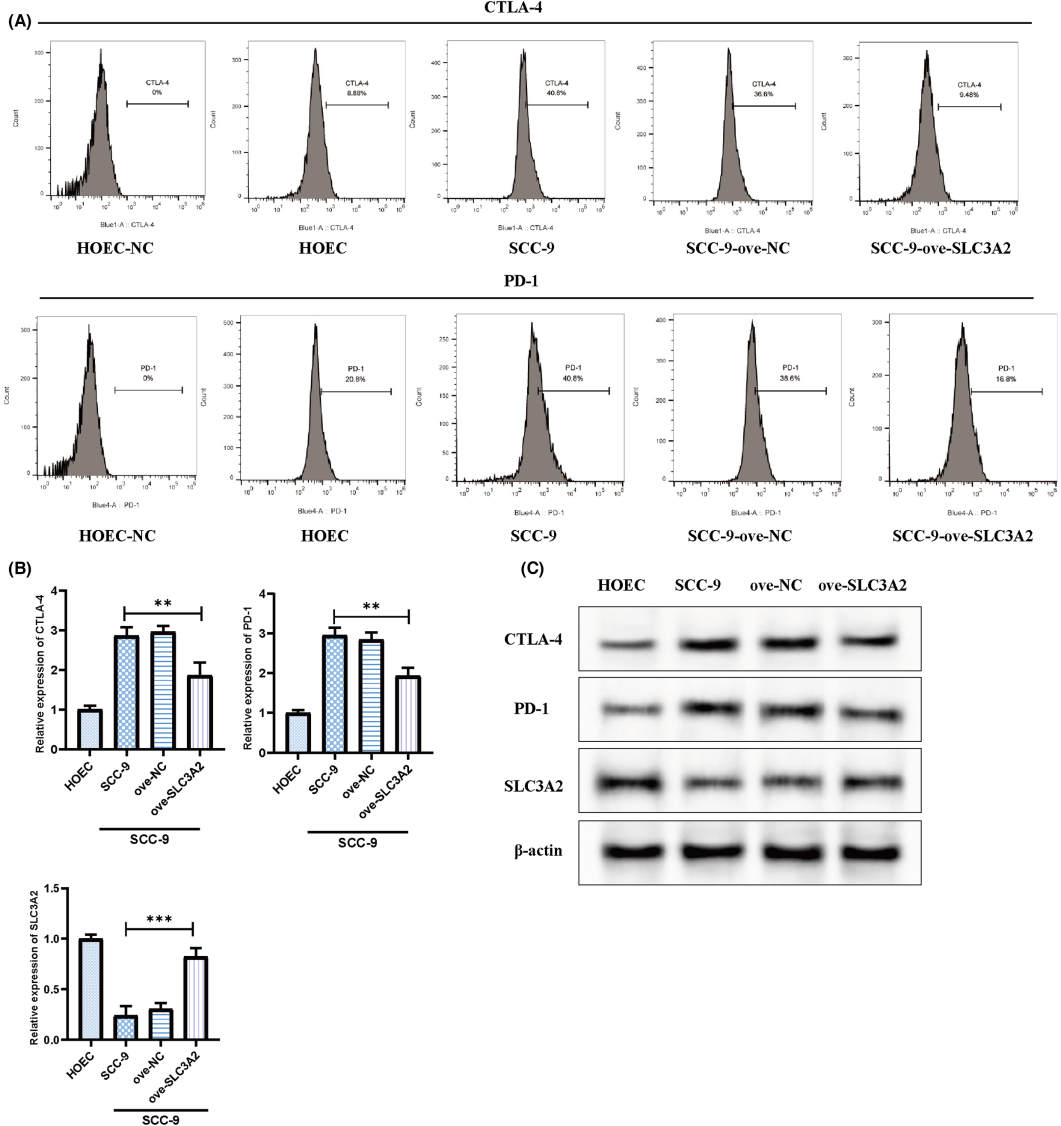
Inhibition of PD‐1 and CTLA‐4 expression by overexpressed SLC3A2. (A) Flow cytometry analysis of PD‐1 and CTLA‐4 expression in different cell groups; (B) qPCR analysis of PD‐1 and CTLA‐4 expression in different cell groups. **p < 0.01; ***p < 0.001; (C) Western blot analysis of related protein expression.

In vitro experiments using over‐SLC3A2 expression showed a decrease in PD‐1 and CTLA‐4 expression in SCC‐9 OSCC cells, as demonstrated by flow cytometry (Figure 8A–D). Western blot analysis also revealed downregulation of PD‐1 and CTLA‐4 protein expression by over‐SLC3A2 (Figure 8E). qPCR results showed a similar decrease in PD‐1 and CTLA‐4 mRNA levels in the over‐SLC3A2 group (Figure 8G). PD‐1 and CTLA‐4 are negative immune regulatory molecules on T cells that can induce immune escape of tumour cells by inhibiting the immune function of T lymphocytes and promote tumour development. These results indicate that overexpression of SLC3A2 effectively suppresses immune escape and enhances immune responses.

## 4 DISCUSSION

OSCC is one of the most common malignant tumours worldwide, with approximately 300,000 new cases reported each year. The highest incidence rates are found in Asia, particularly in Southeast Asia. The main risk factors for OSCC include smoking, alcohol consumption and human papillomavirus (HPV) infection.^14^ Current treatment options for OSCC mainly include surgical resection, radiation therapy and chemotherapy, among other comprehensive approaches.^15^ However, due to late‐stage diagnosis with local invasion and lymph node metastasis, the effectiveness of surgical resection is limited, and there are also limitations in terms of indications and surgical risks. Radiation therapy and chemotherapy can be used as adjuvant or alternative treatments, but the recurrence rate remains high.^16^ Therefore, further research and development of innovative treatment strategies are necessary to improve the therapeutic outcomes for OSCC.

Our study revealed that the genes SLC3A2 and SLC7A11, which are associated with disulphide death, are enriched in tongue squamous cell carcinoma (TSCC). In the GSE37991 dataset, we identified five DEGs related to disulphide death, including SLC3A2, SLC7A11, RPN1, GYS1 and NDUFS1. Disulphide death is a novel form of cell death reported by Professor Ganboyi and his team in the journal *Nature Cell Biology*. It occurs when cancer cells overexpress solute carrier family seven member 11 (SLC7A11), also known as xCT, leading to abnormal accumulation of disulphides (such as cysteine) in the cell due to insufficient NADPH supply under glucose starvation conditions, ultimately resulting in cell death.^8^ Metabolic reprogramming is a crucial hallmark of cancer, often leading to increased uptake of key nutrients such as glucose and glutamine for biosynthesis and bioenergetic processes.^17^ Cancer cells achieve this by upregulating transport proteins for glucose and amino acid uptake. Accordingly, under restricted glucose or amino acid conditions, some cancer cells undergo cell death, while normal cells can survive. This nutrient dependency provides a potential target for cancer therapy through metabolic vulnerabilities.^18^ Professor Ganboyi's research demonstrates that elevated expression of SLC7A11 promotes metabolic vulnerability‐induced disulphide death in cancer cells, which could be an effective strategy for tumour treatment. However, the impact of SLC3A2 expression on OSCC has not been studied yet.

In addition, our study also identified NDUFS1, RPN1 and SLC3A2 as disulphide death genes with good diagnostic significance in TSCC datasets GSE37991 and GSE25099, as indicated by the area under the curve (AUC) greater than 0.70. Currently, most research focuses on the impact of SLC7A11 in TSCC, with no relevant studies on the expression of disulphide death genes NDUFS1, RPN1 and SLC3A2 in TSCC. Furthermore, it has been found that in TSCC, miR‐375 targets SLC7A11, regulating the proliferation and invasion of TSCC cells. Overexpression of miRNA‐375 can inhibit the growth and invasion of TSCC cells by targeting and suppressing SLC7A11 expression, demonstrating the close association between high SLC7A11 expression and TSCC.^18^, ^19^ Therefore, our study provides a ground‐breaking exploration into the molecular mechanism by which downregulation of SLC3A2 expression mediates immune escape and accelerates disulphide death in tumour cells, further advancing the progression of TSCC.

Lastly, our in vitro cell experiments revealed that SLC3A2 mRNA expression levels were higher in the OSCC cell line SCC‐9 compared to normal oral epithelial cells. Overexpression of SLC3A2 significantly inhibited the proliferation of SCC‐9 cells. Moreover, flow cytometry, qPCR and Western blotting results showed that overexpression of SLC3A2 reduced the expression of PD‐1 and CTLA‐4 in the OSCC cell line (SCC‐9). It has been discovered that SLC3A2 plays a crucial role in tumour occurrence and development by participating in glycine transport and regulating extracellular matrix structure.^13^ Studies have also shown a close association between SLC3A2 expression and clinical malignant features such as tumour invasion, metastasis and drug resistance.^20^ Additionally, targeted inhibition of SLC3A2 expression or function has been found to suppress the proliferation and invasive abilities of tumour cells and increase sensitivity to chemotherapy drugs.^21^ These research findings suggest that SLC3A2 may serve as a potential therapeutic target for tumours. In conclusion, the expression alterations of SLC3A2 play a significant role in the growth and development of OSCC through their interaction with the immune response. Future studies will need to delve deeper into the mechanisms underlying the interplay between SLC3A2 expression alterations and the immune response to develop new methods for treating OSCC.

In summary, decreased expression of SLC3A2 in OSCC can induce immune escape of tumour cells by inhibiting the immune function of T lymphocytes, thereby accelerating the progression of OSCC. The expression levels of SLC3A2 can reflect the status of immune response and may serve as a potential biomarker for OSCC. Therefore, it has the potential to become an important research direction in the field of OSCC therapy in the future.

## AUTHOR CONTRIBUTIONS

**Jiang En Wu:** Project administration (equal). **You Li:** Writing – original draft (equal). **Jun Hou:** Writing – review and editing (equal).

## CONFLICT OF INTEREST STATEMENT

The research was conducted in the absence of any commercial or financial relationships that could be construed as a potential conflict of interest.

## Data Availability

The data that support the findings of this study are available on request from the corresponding author. The data are not publicly available due to privacy or ethical restrictions.
